# Divalent metal modulation of Japanese flounder (*Paralichthys olivaceus*) purinergic P2X7 receptor

**DOI:** 10.1002/2211-5463.12375

**Published:** 2018-01-23

**Authors:** Carolina Paredes, Shuo Li, Xiaoli Chen, Claudio Coddou

**Affiliations:** ^1^ Department of Biomedical Sciences Faculty of Medicine Universidad Católica del Norte Coquimbo Chile; ^2^ Tianjin Key Laboratory of Animal and Plant Resistance College of Life Sciences Tianjin Normal University China

**Keywords:** ATP, divalent metals, Japanese flounder, *Paralichthys olivaceus*, purinergic receptor P2X7

## Abstract

*Paralichthys olivaceus* P2X7 receptor (poP2X7R) is a recently identified as a P2X7 purinergic receptor involved in innate immunity of the Japanese flounder *Paralichthys olivaceus*. Divalent metals are allosteric modulators of mammalian P2XRs, but there is no information for fish P2XRs. Here, we characterized the effects of divalent metals on poP2X7R channel activity by electrophysiology and molecular biology techniques. Copper, zinc and mercury inhibited poP2X7R‐mediated currents with different maximal inhibition potency, while cadmium had no effect on poP2X7R activity. Mercury‐induced inhibition was irreversible, but the inhibitory effects of copper and zinc were reversed after washout. Cooper and zinc also reduced poP2X7R‐mediated interleukin‐1 mRNA production. These findings suggest that divalent metals have potential effects on the Japanese flounder innate immune response through modulation of poP2X7R activity.

**Abbreviations**
HKMs: head kidney macrophages
IL‐18: interleukin 18
IL‐1β: interleukin 1 beta
P2XRs: purinergic P2X receptors
PBLs: peripheral blood leukocytes
poP2X7R: *Paralichthys olivaceus* P2X7 receptor
rP2X7R: Rat P2X7 receptor

Extracellular ATP and related nucleotides participate in a variety of signaling processes via the activation of different classes of membrane receptors, which are termed purinergic receptors. These receptors include the metabotropic P2Y receptors (P2YRs) and ionotropic purinergic P2X receptors (P2XRs), which are responsible for the purinergic signaling in immune responses, reproduction, neurotransmission and other biological processes 1, 2, 3]. P2XRs are ATP‐gated ion channels with a unique structure; each subunit has two membrane spanning regions with both C‐ and N‐terminal ends facing the cytosol and three of these subunits conform the functional channels that can be either homomeric or heteromeric 1, 4, 5.

Among the P2XR subfamily, the P2X7R is of particular interest, because of its wide expression in immune cells such as macrophages, dendritic cells, monocytes, B‐lymphocytes and T‐lymphocytes, and its involvement in immunological processes 6. The P2X7R needs high extracellular ATP concentrations to be activated (in the millimolar range), shows an increase in currents instead of desensitization when exposed to long ATP applications in contrast to other P2XRs 1, 5. Human and rat P2X7Rs are very sensitive to divalent cations that inhibit the channel's activity in the low micromollar range 1, 7, 8. When activated, the P2X7R plays an important role in the innate immune responses through an increase in the production of proinflammatory cytokines interleukin (IL)‐18 and IL‐1β 9, induction of apoptosis 10, generation of reactive oxygen and nitrogen intermediates 11 and stimulation of phagosome–lysosome fusion 12. These particular roles of the P2X7R are in part related to a unique cysteine‐rich region in the C‐terminal domain that in conjunction with other intracellular domains has been postulated to be important for the interaction of the receptor with other immune response‐related molecules 13. Although the gating and physiological roles of mammalian P2X7Rs have been extensively investigated, there is much less information in lower vertebrates such as in fish. Lopez‐Castejon *et al*. 14 have characterized the pharmacological properties of P2X7R from the gilthead seabream (*Sparus aurata* L.) by electrophysiological recordings. Recently, we have identified a functional P2X7R homolog from the teleost fish Japanese flounder (*Paralichthys olivaceus*) ortholog, termed poP2X7R, and studied its engagement in the Japanese flounder innate immune response 15. However, the effects of divalent cations on P2X7Rs in teleost are still lacking.

In the present work, we investigated the effects of four divalent metals including zinc, copper, cadmium and mercury on the poP2X7R activity using electrophysiology and molecular biology techniques. We found that although some of these metals can consistently inhibit the channel‐mediated activity, the fish P2X7 receptor is much more resistant to metal‐induced inhibition, as compared to its mammalian counterparts, and this modulation may provide a mechanism in regulation the P2X7R‐mediated innate immune response in the Japanese flounder.

## Materials and methods

### Ethics statement

All experiments were conducted in accordance with the NIH guidelines for the care and use of experimental animals and the studies were specifically approved by the animal care and use committees of Tianjin Normal University and Universidad Catolica del Norte.

### Animals and maintenance

Japanese flounders (*P. olivaceus*) were purchased from a local farm in Dagang, Tianjin, China, transported to the laboratory and maintained in aerated running seawater aquaria at 21 °C for 2 weeks before experiments. Animals were fed with a commercial pellet diet twice at a ratio of 2% body weight per day and only healthy animals were selected in experiments. For tissue collection, *P. olivaceus* was euthanized with 0.25 g·L^−1^ tricaine methane sulfonate (Sigma‐Aldrich, St. Louis, MO, USA) and the individual tissue was then dissected aseptically. For the experiments with oocytes, females of the African frog, *Xenopus laevis* were kept in the animal facility of the Universidad Catolica del Norte. A segment of the ovary was surgically removed under anesthesia (benzocaine, 0.05%), in order to extract the oocytes.

### Oocyte injection and electrophysiology

Oocytes were manually defolliculated and incubated 30 min with type III collagenase as previously described 16. The pIRES‐EGFP/poP2XR7 plasmid was generated as previously described 15. Oocytes were injected intranuclearly with 4 ng Japanese flounder or rat P2X7R cDNA. After 12–48 h incubation in Barth's solution (in mm; 88 NaCl, 1 KCl, 2.4 NaHCO_3_, 10 HEPES, 0.82 MgSO_4_, 0.33 Ca(NO_3_)_2_, 0.91 CaCl_2_; pH 7.5) supplemented with 10 U·L^−1^ penicillin/10 mg streptomycin and 2 mm pyruvate, oocytes were clamped at −70 mV using the two‐electrode voltage‐clamp configuration with an OC‐725C clamper (Warner Instruments Corp., Hamden, CT, USA). ATP‐gated currents were recorded following regular ATP applications. The recordings were performed either in Barth's (in mm; 88 NaCl, 1 KCl, 2.4 NaHCO_3_, 10 HEPES, 0.82 MgSO_4_, 0.33 Ca(NO_3_)_2_, 0.91 CaCl_2_; pH 7.5) or in a low‐divalent (LD) containing media (in mm; 91 NaCl, 1 KCl, 0.5 CaCl_2_, 0.1 MgCl_2_, 10 HEPES; pH 7.5). Noninjected oocytes did not evoke currents when exogenous ATP was applied. ATP and divalent metals were dissolved in Barth's or LD media and perfused using a peristaltic pump operating at a constant flow of 2 mL·min^−1^. Metal concentration–response curves were performed by preapplying the metal solution for 90 s and then coapplying the same solution with 1 mm ATP for 30 s. Metal concentrations ranged from 1 to 300 μm. Washout periods ranged from 5 to 15 min depending on metal concentration.

### Primary cells preparation

Japanese flounder head kidney primary cells were prepared as described by Li *et al*. 15, 17. Peripheral blood was collected from the caudal vein of individual fish with a 10‐mL heparinized syringe. These cells were then used for further isolation of Japanese flounder head kidney macrophages (HKMs) and peripheral blood leukocytes (PBLs) by discontinuous Percoll density (1.020/1.070 and 1.070/1.077, respectively; GE Bio‐Sciences, Pittsburgh, PA, USA) gradient centrifugation. After centrifugation at 400 ***g*** for 30 min at 4 °C, the white interface fraction was collected and washed three times with cold PBS. The isolated HKMs and PBLs were then resuspended in culture medium [RPMI 1640 supplemented with 10% FBS, and 1% penicillin–streptomycin liquid (Thermo Scientific, Rockford, IL, USA)] and cultured at 21 °C overnight before experimentation.

### PoP2RX7‐mediated IL‐1β gene expression

To examine the involvement of poP2X7R in ATP‐evoked cytokine gene expression, overnight cultured Japanese flounder HKMs or PBLs at a density of 1.0 × 10^7^/well were preincubated with or without 200 μm cooper or zinc for 2 h and then treated with 1 mm ATP for 30 min to activate poP2X7R. The treated cells were finally incubated with normal culture medium for 2 h and used for RNA isolation. RNA was then purified using a RNeasy mini kit (QIAGEN, Germantown, MD, USA) and transcribed into cDNAs. The gene expression changes of *IL‐1*β were determined by quantitative real‐time PCR.

## Results

### Effects of divalent metals on poP2X7R activity

**Figure 1:**
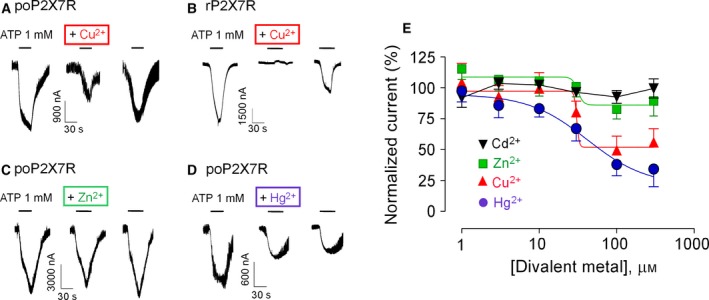
Modulation of poP2X7R channel activity by divalent metals. (A–D) Representative recordings from single oocytes expressing the poP2X7R (A, C and D) or the rat P2X7R (rP2X7R, B) showing macroscopic currents evoked by the application of 1 mm of ATP alone (left tracings) or after a 90 s of preapplication of 100 μm of the metal (middle tracings) and their respective washouts (right tracings). For the experiments with rP2X7R, 10 μm of Cu^2+^ was used. The divalent metals tested were copper (Cu^2+^, A,B), zinc (Zn^2+^, C) and mercury (Hg^2+^, D). (E) Summary of divalent metals concentration–response experiments (1–300 μm) on 1 mm of ATP‐evoked currents (*n* = 5–7). The metals are the same showed in the tracings plus cadmium (Cd^2+^).

In order to test the effects of copper, zinc, cadmium and mercury on the activity of the poP2X7R, we expressed this receptor channel in *X. laevis* oocytes and measured the currents gated by this channel with the two‐electrode voltage‐clamp technique. In poP2X7R‐expressing oocytes, 1 mm of ATP induced slow cationic currents (Fig. 1A), a hallmark of P2X7R, and the preapplication for 90 s of 100 μm copper (Cu^2+^) and subsequently coapplication with 1 mm of ATP inhibited the currents by 50% (Fig 1A); this inhibition was partially recovered after a 15 min washout and total recovery was obtained only after 2–3 washouts (not shown). We next tested several copper concentrations and revealed that the poP2X7R was resistant to copper in the range of 1–10 μm, but 30–300 μm of copper inhibited the ATP‐evoked currents concentration dependently with an estimated IC_50_ (half maximal inhibitory concentration) of 30.5 μm and a maximal current inhibition of ~ 53% (Fig. 1A,E; Table 1). In contrast, the rat P2X7 receptor (rP2X7R) was completely inhibited by 10 μm of copper (Fig. 1B), suggesting that the poP2X7R is more resistant to this divalent metal.

**Table 1:** Divalent metal IC_50_ and maximal inhibition of P2X7R‐mediated currents

Metal	IC_50_ (μm)	Maximal inhibition (%)	*n*
Cu^2+^	30.5 ± 5.6	52.8 ± 14.5	5–6
Zn^2+^	31.6 ± 14.3	16.0 ± 9.5	6
Hg^2+^	29.8 ± 10.2	65.8 ± 6.3	4–6
Cd^2+^	n.e	n.e	7

n.e., no effect.

Next, we tested the effects of zinc (Zn^2+^) on the poP2X7R, using a similar approach to that applied with copper. When preapplied for 90 s and coapplied with ATP, 30–300 μm of Zn^2+^ slightly inhibited the ATP‐evoked, with an estimated IC_50_ of ~ 32 μm and a maximal inhibition of 16% (Fig. 1C,E; Table 1). In the case of cadmium (Cd^2+^), we found no modulatory effects (no inhibition nor potentiation) at 1–300 μm (Fig. 1E and Table 1), suggesting that the poP2X7R is completely resistant to cadmium. In a final set of electrophysiology experiments, we tested the effects of mercury (Hg^2+^). Low mercury concentrations (1–3 μm) did not induced any significant effect, but 10–300 μm of mercury inhibited the ATP‐evoked currents in a concentration‐dependent manner (Fig. 1D,E; IC_50_ and maximal inhibition values are shown in Table 1). In contrast to the other divalent metals tested, the mercury‐induced inhibition was irreversible and recovery of the original response was never achieved, independent of the washout time (see recordings in Fig. 1D); for that reason, in most experiments with 10–300 μm of mercury, one oocyte was used in per dose.

### Effects of copper and zinc on the poP2X7R‐mediated expression of IL‐1β

**Figure 2:**
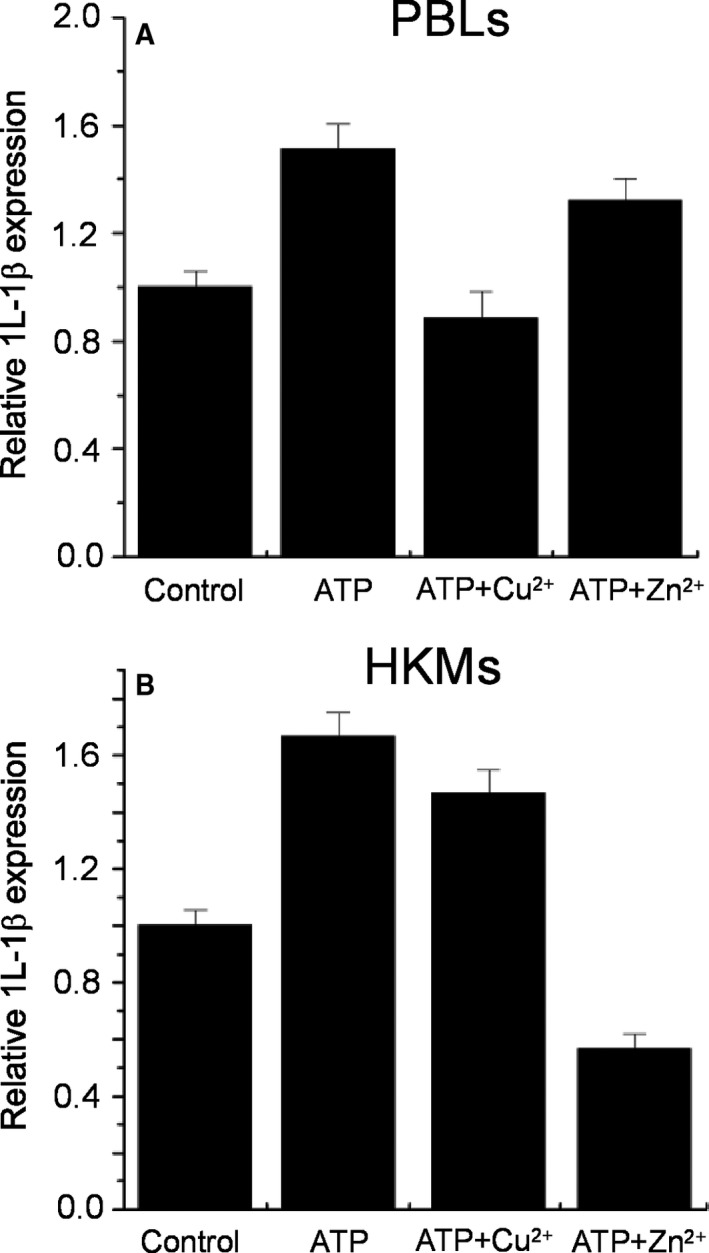
Effects of Cu^2+^ and Zn^2+^ on poP2X7R‐mediated IL‐1β gene expression. Overnight cultured Japanese flounder PBLs (A) or HKMs (B) were incubated with 1 mm of ATP alone or together with 200 μm of Cu^2+^ or Zn^2+^. After treatment, total RNA was extracted from the cells and the levels of IL‐1β gene expression were measured by qPCR (*n* = 3).

As we have previously showed, the poP2X7R mediates the production of several cytokines in the Japanese flounder immune cells 15. To evaluate the involvement of divalent metal on P2X7R‐mediated immune response in the Japanese flounder, we tested the effects of copper and zinc metals on extracellular ATP‐induced IL‐1β expression by qRT‐PCR in the PBLs and HKMs, two types of Japanese flounder immune cells that endogenously express poP2X7R. In PBLs, 1 mm of ATP increased 1.5‐fold IL‐1β gene expression, which could be completely abolished by preincubation with 200 μm of copper (Fig. 2A). In contrast, 200 μm of zinc was unable to prevent the ATP‐induced IL‐1β expression (Fig. 2A). On the other hand, in the HKMs, 200 μm of copper was unable to inhibit the ATP‐induced increase in IL‐1β gene expression, but in this cell‐type, zinc decreased the ATP‐induced IL‐1β gene expression (Fig. 2B).

## Discussion

It is known that divalent metals can regulate the functions of several proteins; in fact, they are key components of hormones, prosthetic enzyme groups and transport proteins 18. Moreover, metals can modulate the activity of several voltage and ligand‐gated ion channels, and therefore, they influence the signaling processes mediated by these channels 1, 18, 19. In the specific case of P2XR channels, divalent metals can exert a variety of effects, including inhibition and potentiation of the channel activity; these effects depends both in the nature of the divalent metal and the receptor subtype 1, 18. Here, we have characterized the effects of four divalent metals, copper, zinc, cadmium and mercury on the channel activity of the purinergic receptor P2X7 (poP2X7R) cloned from the Japanese flounder *P. olivaceus*, and tested their effects on the poP2X7R‐mediated innate immune responses.

From the four divalent metals tested, copper, zinc and mercury exerted an inhibitory modulation of the poP2X7R‐mediated currents, but cadmium showed no effect on the activity of this receptor. Moreover, the divalent metals that inhibited the receptor exhibited similar potencies but different efficacies, suggesting that the receptor can discriminate between divalent metals and their effects are specific. For example, the maximal inhibition induced by zinc was only about a 16%, a value significantly lower than the maximal inhibition induced by copper (52%) and mercury (65%). The effects of copper and zinc were reversible as it was possible to recover the original response after 1–2 washouts; in contrast, mercury‐induced inhibition was irreversible and with concentrations over 30 μm we have to use only one oocyte per dose, highlighting the toxic nature of this metal. This observation suggests that similar to other proteins 20, mercury binds irreversibly to the poP2X7R, changing its properties and impairing its physiological functions. In the specific case of purinergic receptors, we have previously reported that the rP2X2R is positively modulated by mercury and reactive oxygen species by an intracellular mechanism 21. Similar to the results found in the present work, the effects of mercury on the rP2X2R are irreversible and modify permanently the gating properties of this receptor channel 21.

**Figure 3:**
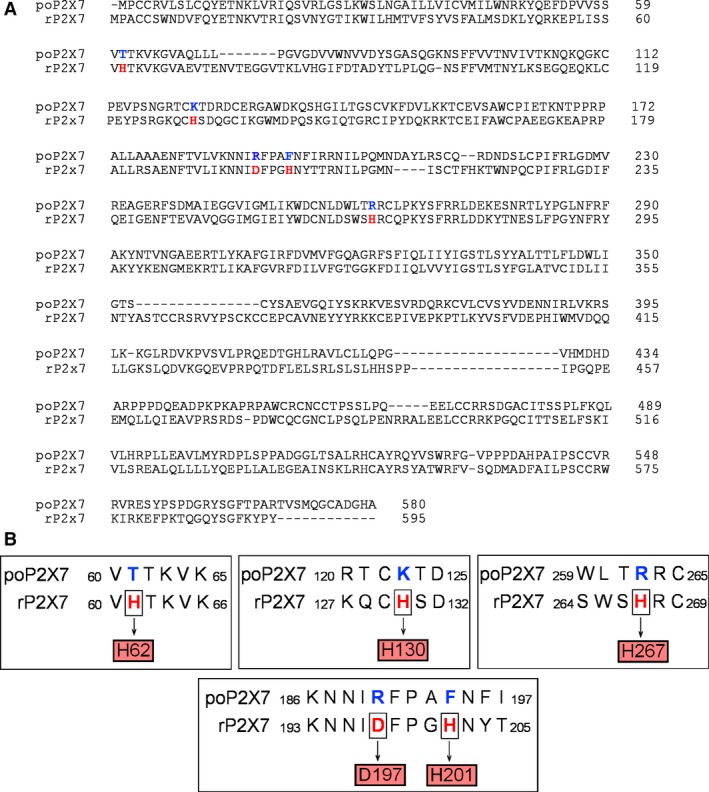
Copper and zinc ion binding sites present in rP2X7R are absent in poP2X7R. A. Alignment of the amino acid sequences of the rat P2X7R (rP2X7R) and its counterpart in the Japanese flounder (poP2X7R). In red are shown the extracellular residues identified as important for copper and zinc inhibition of the rat P2X7R. The corresponding residues in *Paralichthys olivaceus* P2X7R are shown in blue. B. Details of the extracellular regions that are important for copper and zinc inhibition in the rP2X7R and the corresponding residues present in the poP2X7R.

One remarkable finding of this study is that although copper and zinc have the same inhibitory effect on the poP2X7R as compared to its mammalian (human, rat and mouse) counterparts, their potencies and efficacies are significantly lower on the Japanese flounder P2X7 receptor. For example, the rat receptor (rP2X7R) shows an IC_50_ for copper of 4.4 μm and a maximal inhibition of 100% 8, 22, 23; in the case of zinc, the reported IC_50_ and maximal inhibition are 78 μm and 90% 8. In the human P2X7R, similar IC_50_ values for copper have been reported 24. In contrast, the poP2X7R is much more resistant to these metals, a feature that could be a consequence of the difference between the marine and terrestrial environments. It is interesting to note that when we compare the regions and residues proposed to form the binding sites for copper and zinc in the rP2X7R 8, 23, the aspartic acid and histidine residues present in the rat receptor are absent in the Japanese flounder, and this can explain the higher resistance of poP2X7R to copper, zinc and other divalent metals (Fig. 3). More specifically, in the rP2X7R, it has been proposed that H62, H130, D197, H201 and H267 (rat numbering) 8, 23 are important for copper and zinc inhibition. Analyzing the poP2X7R sequence we found that only T62 and F194 are able to coordinate divalent metals since in the other potential binding sites positively charged amino acids are present (Fig. 3). We hypothesize that these distinct features in the Japanese flounder P2X7R can be a result to an evolutionary process in order to confer resistance to divalent metals in an environment with higher potential exposure.

After characterization of divalent metal effects on poP2X7R activity, we next examined the functional consequences of these modulatory effects on P2X7R‐mediated innate immune response in the Japanese flounder. The role of extracellular ATP and purinergic receptors in inflammation and immunity has been consistently demonstrated in mammals 13. Although there are several ATP‐gated P2XR subtypes expressed in immune cells (e.g. P2X1R, P2X4R or P2X6R), the P2X7R is the only member that has been consistently demonstrated to play a role in immune responses, specifically in regulating cytokine production and release 13. We previously reported that this role is conserved in the Japanese flounder, i.e. activation of poP2X7R can induce an increased gene expression of the proinflammatory cytokines IL‐1β and IL‐6 in the Japanese flounder head kidney primary cells 15. Moreover, we have demonstrated the release of ATP by connexin43 and pannexin1 channels in the Japanese flounder immune cells under inflammatory conditions, further supporting the role of extracellular ATP and its associated membrane receptors in fish innate immune response 25, 26. Thus, it was important to test if divalent metal administration could impact the poP2X7R‐mediated cytokine synthesis. To this aim, we assessed the effects of copper and zinc on the IL‐1β expression in *P. olivaceus* immune cells (Fig. 2). Interestingly, we found that copper but not zinc inhibited the ATP‐induced increase in IL‐1β expression in the PBLs but not in the HKMs in which zinc inhibited the ATP‐induced cytokine expression, but not copper. This result may be explained by a recent publication of our group, in which we found that PBLs predominantly express poP2X7R, but in contrast in HKMs the dominant expressed purinergic receptor is the P2X2R 27. At the moment, we have not explored the effects of divalent metals on the poP2X2R but we can infer that this receptor could be more susceptible to zinc modulation, future experiments will help to clarify this point.

In summary, we have characterized for the first time the effects of divalent metals on the activity of the poP2X7R, an ATP‐gated ion channel that is involved in the innate immune response in teleost fish *P. olivaceus*. Our findings also pointed out a possible modulatory role of heavy metals from marine pollution on P2X7R‐mediated fish immune response.

## Author contributions

CC and SL conceived and supervised the study; CC and SL designed experiments; CP and XC performed experiments; CP, XC, SL and CC analyzed experiments; CC and SL wrote the manuscript.
